# Integrated Metagenomic and Metatranscriptomic Analysis Reveals Microbiome Differences Between Bisexual and Parthenogenetic Populations of *Haemaphysalis longicornis*

**DOI:** 10.3390/microorganisms14102345

**Published:** 2026-10-14

**Authors:** Wen-Jie Zhu, Yuchao Zhang, Meiqi Mu, Tingwei Pei, Han Wang, Xiujie Liang, Yunsheng Tang, Chunyuan Wang, Yingying Dai, Zhen Wang, Zifeng Liu, Zhijun Yu, Tianhong Wang

**Affiliations:** 1 Department of Biochemistry and Molecular Biology, College of Pharmacy, Hebei University of Chinese Medicine, Shijiazhuang 050200, China; zhuwenjie99566@163.com (W.-J.Z.); 13483316407@163.com (Y.Z.); 19061842520@163.com (M.M.); 2 Department of Critical Care Medicine, The Second Hospital of Hebei Medical University, Shijiazhuang 050000, China; 3 Hebei Key Laboratory of Animal Physiology, Biochemistry and Molecular Biology, College of Life Sciences, Hebei Normal University, Hebei Collaborative Innovation Center for Eco-Environment, Shijiazhuang 050024, China; ptw0927@163.com (T.P.); 15503058207@163.com (H.W.); liangxiujie2024@163.com (X.L.); tys14027@163.com (Y.T.); 18920547228@163.com (C.W.); 18230269211@163.com (Y.D.); 18833197638@163.com (Z.W.); 15833933529@163.com (Z.L.)

**Keywords:** bisexual and parthenogenetic, *Haemaphysalis longicornis*, metagenomics, metatranscriptomics, RNA/DNA relative activity

## Abstract

The tick *Haemaphysalis longicornis* is an important vector of human and animal pathogens in East Asia that comprises both bisexual (Bis) and parthenogenetic (Par) populations. While previous studies have documented differences between these reproductive groups in genome ploidy, host transcription, and vector capacity, it remains unclear whether reproductive strategy is accompanied by systematic divergence in the tick-associated microbiome, including microbial community composition, transcriptional activities, and functional outputs. Here, we profiled laboratory-maintained Bis and Par populations using metagenomic (MG) and metatranscriptomic (MT) sequencing to integrate community composition, core taxa, functional potential, and RNA/DNA relative activity (RNA abundance normalized to corresponding DNA abundance). Par populations exhibited higher MG alpha diversity, whereas Bis populations showed higher MT alpha diversity, indicating a decoupling between the microbial DNA reservoir and the active RNA transcript community. MG functional analysis revealed that Par populations possessed a higher potential for environmental sensing, membrane transport, biofilm formation, porphyrin metabolism, and iron acquisition. Conversely, Bis populations exhibited enhanced potential in biotin metabolism, lipoic acid metabolism, and oxidative phosphorylation. MT analysis identified significant differences in a limited number of species and CAZy families, while selected KEGG pathways showed no significant variance, suggesting restricted rather than systemic transcriptional differentiation. By integrating DNA and RNA profiles, we classified 279 shared species into four activity states, revealing higher RNA/DNA relative activity ratios for thiamine metabolism in Par, and for biofilm formation, flagellar assembly, sulfur metabolism, and fatty acid degradation in Bis. Overall, Bis and Par populations exhibit distinct differences in their microbial reservoirs, transcript compositions, and relative transcriptional activities, providing critical microbiome insights into the symbiosis and vector adaptation associated with reproductive strategies in *H. longicornis*.

## 1. Introduction

Ticks are critical medical arthropod vectors, second only to mosquitoes in public health relevance, transmitting diverse viruses, bacteria, rickettsiae, spirochetes, and protozoa [1,2]. Over the past two decades, the spectrum, geographic distribution, and public health burden of tick-borne infections in mainland China have continuously expanded [3,4]. Amid the ongoing discovery and reassessment of agents such as severe fever with thrombocytopenia syndrome virus (SFTSV), spotted fever group rickettsiae, *Anaplasma*, *Ehrlichia*, and *Babesia*, tick-borne diseases have become a vital focus of zoonotic disease surveillance [5]. Beyond the targeted detection of known pathogens, integrated omics approaches are uncovering complex microbial ecosystems within ticks, encompassing symbionts, opportunistic pathogens, and environmental microorganisms [6]. This expanded perspective is crucial for clarifying mechanisms of pathogen maintenance, transmission risks, and vector expansion.

*Haemaphysalis longicornis* Neumann, 1901 is a widespread tick species in East Asia that parasitizes livestock, wildlife, and humans, acting as a key vector for SFTSV and other agents [7,8]. This species uniquely comprises coexisting bisexual (Bis) and parthenogenetic (Par) reproductive populations [9]. Recent work further indicates that the triploid genome state of Par ticks is associated with their rapid geographic expansion and enhanced vector capacity [10]. *H. longicornis* has also established invasive populations in multiple US states, attracting sustained attention [11]. Linking the differences between these reproductive forms with their microbiome structure and function may therefore help generate hypotheses about host–microbiome interactions concerning parthenogenetic expansion, nutritional adaptation, and pathogen carriage.

The tick microbiome functions beyond a mere list of pathogens, acting as a dynamic ecosystem intertwined with tick nutrition, development, reproduction, immune regulation, and pathogen transmission [12,13]. For example, *Coxiella*-like endosymbionts can synthesize essential B vitamins to compensate for nutritional deficits in obligately blood-feeding arthropods [14], while the tick gut microbiota can modulate the local environment required for pathogen colonization [13,15]. Previous studies have characterized the microbiome composition, functional annotations, and virome diversity of *H. longicornis* and other ticks [16,17,18,19,20], while also beginning to resolve tissue-specific microbiomes and signals related to pathogens [21]. However, most existing literature remains centered on DNA-based detection or carriage profiles. While metagenomics (MG) evaluates community members and their coding potential, metatranscriptomics (MT) provides a more accurate reflection of transcript composition at the time of sampling; divergence between the two can expose abundant but weakly transcribed reservoir members, or conversely, identify highly active members with low DNA abundance but strong RNA representation [22,23,24].

Accordingly, we used Bis and Par populations maintained in the laboratory as a comparative model to characterize microbial diversity, taxonomic composition, core genera, functional potential and transcriptional activity, and used RNA/DNA ratios to evaluate relative transcriptional activity. By integrating MG and MT data, we sought to identify taxonomic and functional features that differences between populations and to distinguish functional potential encoded in DNA from its active representation in RNA, thereby providing molecular evidence for microbiome composition and functional differentiation between the reproductive populations of *H. longicornis*.

## 2. Materials and Methods

### 2.1. Tick Populations, Rearing Conditions, and Experimental Design

We utilized two laboratory-maintained populations of *H. longicornis*: a bisexual (Bis; B1–B5) and a parthenogenetic (Par; P1–P5) population. To enable matched feature comparisons, identical sample identifiers were used across both MG and MT datasets, comprising five independent biological replicates per population for each omics layer. Each biological replicate consisted of 5 unfed female ticks (2–4 weeks post-molt) pooled into a single sample. Prior to tick placement, rabbit ear skin was wiped with 75% ethanol. Ticks from different populations were fed on separate rabbits, and no rabbit was reused across populations or feeding batches. During non-parasitic stages, ticks were housed in an artificial climate chamber at 26 ± 1 °C with 85% relative humidity under a 16-h light/8-h dark photoperiod. Both populations had been continuously cultured in the laboratory for over 10 generations.

### 2.2. Extraction of Nucleic Acids, Library Preparation, and Sequencing

Prior to nucleic acid extraction, ticks from each pooled sample were subjected to surface sterilization to remove external contaminants. Briefly, ticks were washed twice with sterile phosphate-buffered saline (PBS), then soaked in 70% ethanol for 30 s, followed by 10% sodium hypochlorite for 30 s, and finally washed twice with ultrapure water. Surface-sterilized ticks were immediately processed for DNA and RNA extraction. Total DNA and total RNA extraction, quality assessment, library construction and high-throughput sequencing for all samples were performed by Biomarker Technologies (Beijing, China). DNA concentration was measured using a Qubit 2.0 fluorometer (Thermo Fisher Scientific, Waltham, MA, USA), and purity was assessed by OD260/280 and OD260/230 ratios using a NanoDrop 2000 spectrophotometer (Thermo Fisher Scientific, Waltham, MA, USA). DNA integrity was verified by 1% agarose gel electrophoresis. RNA concentration was quantified with a Qubit RNA assay, and purity was measured by NanoDrop (Thermo Fisher Scientific, Wilmington, DE, USA). RNA integrity and RIN values were determined using an Agilent 2100 Bioanalyzer (Agilent Technologies, Santa Clara, CA, USA). Samples were considered suitable for library construction if DNA OD260/280 was between 1.8 and 2.0 and RNA RIN was ≥7.0. MG libraries were prepared using the NEBNext Ultra II DNA Library Prep Kit for Illumina (NEB, Ipswich, MA, USA) following the manufacturer’s instructions. Briefly, qualified genomic DNA was fragmented by sonication to a target insert size of approximately 350 bp, followed by end repair, 3′-adenylation and ligation of Illumina sequencing adapters. Ligated products of the appropriate length were size-selected, purified and amplified by PCR to generate sequencing libraries. For MT library construction, total RNA quality was first evaluated, and ribosomal RNA (rRNA) was depleted using the Illumina Ribo-Zero Plus rRNA Depletion Kit, which targets both eukaryotic host rRNA (18S, 28S) and prokaryotic microbial rRNA (16S, 23S). The rRNA-depleted total RNA was fragmented and reverse-transcribed into complementary DNA (cDNA), followed by end repair, 3′-adenylation, adapter ligation, fragment selection (target insert size approximately 350 bp), purification and library amplification. The efficiency of rRNA depletion was evaluated post-sequencing by confirming that rRNA-derived reads accounted for only a minor fraction of the total MT data. After quality control, both MG and MT libraries were sequenced on an Illumina NovaSeq 6000 platform (Illumina, San Diego, CA, USA) in paired-end mode with a read length of 2 × 150 bp. The resulting MG data ranged from 20.42 to 25.23 Gb per sample, and MT data ranged from 9.65 to 12.20 Gb per sample.

### 2.3. Sequence Quality Control and Removal of Host Reads

Raw MG and MT reads were processed with fastp v0.23.2 to remove adapters, along with low-quality and ambiguous reads [25]. Host-derived sequences were then filtered out by aligning against host reference genomes using Bowtie2 v2.4.5 [26]. The Bis and Par reads were aligned against reference genomes GCA_013339765.2 and GCA_055474425.1, respectively. Clean bases, clean reads, GC content, Q20 and Q30 were recorded after quality control. The fraction retained after host removal was calculated as no-host clean bases/clean bases × 100%. The mapped rate of the non-redundant MT gene set was also recorded. Complete quality control metrics for individual samples are provided in Table S1, with group means summarized in Table 1.

### 2.4. Sequence Assembly, Gene Prediction, and Construction of Nonredundant Gene Catalogs

MG reads remaining after host removal were assembled with MEGAHIT v1.2.9 [27], and MT reads remaining after host removal were assembled into transcripts with Trinity v2.15.1 [28]. Sequences shorter than 300 bp were removed from both assemblies. MT assembly quality was additionally assessed with QUAST v5.2.0 [29]. For the MT PHI-base annotations, entries assigned to multiple phenotype categories were divided equally among their respective labels and then summed by category. Open reading frames were predicted with MetaGeneMark v3.26 [30] (specifically utilizing version v3.26 for the MT data). Predicted proteins were clustered using MMseqs2 v14-7e284 [31] to construct non-redundant gene sets, applying a sequence identity threshold of 90% and a coverage threshold of 80%. The final MG non-redundant gene set contained 833,638 genes with a total length of 179,389,236 bp, a mean length of 215 bp and a length range of 102–5178 bp. The MT non-redundant gene set contained 69,985 genes with a total length of 35,692,533 bp, a mean length of 510 bp and a length range of 102–19,578 bp.

### 2.5. Taxonomic Profiling, Abundance Estimation, and Microbial Community Diversity

Non-redundant gene sets constructed from predicted proteins (see Section 2.4) were used as the reference catalog for all taxonomic annotation and read abundance calculations. High-quality reads from each sample were then mapped back to the non-redundant gene set to generate read count tables. For taxonomic annotation, the non-redundant gene sets were searched against the NCBI non-redundant protein (NR) database (accessed in March 2026) using BLASTP v2.13.0 with an e-value cutoff of 1 × 10^−5^ and a query coverage cutoff of 50%. Gene abundance was normalized as reads per kilobase per million mapped reads (RPKM) to correct for gene length and sequencing depth, since gene length and read count are correlated in shotgun sequencing data. Relative abundances at the phylum, genus and species levels were calculated by dividing each taxon’s RPKM by the total annotated RPKM within each sample and expressed as percentages; these relative abundance tables were used for all downstream diversity analyses. Alpha diversity was assessed using the Chao1, Shannon, Simpson and Pielou evenness indices based on the species-level relative abundance table. Beta diversity was evaluated by principal coordinate analysis (PCoA) based on Bray–Curtis distances calculated from the species-level relative abundance data. Analysis of similarity (ANOSIM) [32] and permutational multivariate analysis of variance (PERMANOVA) [33] were both performed with 999 permutations to test for compositional differences between Bis and Par populations. ANOSIM was used because it is robust to non-normality and provides a simple R package vegan v2.6-4 statistic for between-group vs. within-group ranking differences, while PERMANOVA was used as a complementary test that partitions distance matrices to partition variance and provides an R^2^ effect size. Multivariate dispersion was assessed using PERMDISP to confirm that significant PERMANOVA results were not driven by differences in within-group dispersion. All tests compared the two reproductive populations (Bis vs. Par) as the sole grouping factor.

### 2.6. Identification of Core Genera, Viral Transcriptional Signals, and Differential Microbial Markers

A core genus was defined as a genus detected in at least four of the five replicates within a population at a relative abundance > 0.001% and with a population mean relative abundance ≥ 0.2%. Genera meeting these criteria in both populations were classified as shared core genera, whereas genera meeting the criteria in only one population were classified as population-specific core genera. The MT viral profile was extracted from the annotation matrix at species level and viral entries were displayed in descending order of the higher group mean. Viral entries were identified from taxonomic assignments against the NR database after host read filtering (Section 2.3). As the present study did not perform de novo viral genome assembly or screen for endogenous viral elements integrated into the tick genome, these entries represent database annotation signals rather than confirmed active infections. Candidate markers related to pathogens and symbionts were identified by LEfSe analysis of the MT abundance matrix at species level [34], using LDA score > 4.0 and nominal *p* < 0.05 as selection criteria.

### 2.7. Functional Annotation and Characterization of Metagenomic Potential

Protein sequences corresponding to the MG and MT non-redundant gene sets were searched against the KEGG database using BLASTP v2.13.0 [35]. MG annotations were used to characterize the functional coding potential of the microbial community, whereas MT annotations were used to describe the relative composition of functional transcripts at the sampling time point. Heatmap values were transformed as log10 (relative abundance (%) + 1 × 10^−4^) and standardized by pathway using z-scores. Z-score standardization is carried out independently for each metabolic pathway, so that the heatmap can intuitively show the relative enrichment or depletion of a given pathway within a sample relative to its mean abundance profile. A positive z-score means the pathway’s reads are higher than the average, whereas a negative z-score means they are lower than the average [36]. CAZy family annotations were generated by HMMER v3.3.2 hmmscan against dbCAN CAZy family hidden Markov models (dbCAN release, accessed March 2026) (e-value < 1 × 10^−5^) [37,38]. PHI-base v4.19 was searched by BLASTP with an e-value cutoff of 1 × 10^−5^ [39], and CARD v4.0.1 (ARO) was searched by BLASTP with the same e-value cutoff [40]. Relative abundances of these functional features were compared between Bis and Par using two-sided Mann–Whitney *U* tests, with Benjamini–Hochberg correction for multiple testing and *q* < 0.05 was considered statistically significant.

### 2.8. Characterization of Metatranscriptomic Taxonomic and Functional Profiles

Using the MT relative abundance matrix at the species level, species were ranked by their mean relative abundance across all samples, and the top 15 species were selected to describe the main transcript sources within the community. Relative abundances were transformed as log10 (relative abundance (%) + 1 × 10^−4^) and standardized by species using row-wise z-scores. A candidate species set was constructed by case-insensitive matching of prespecified taxon-name keywords related to tick symbionts and reported or opportunistic pathogens. Among the 63 identified matches, the 12 species with the highest overall mean MT relative abundance were selected for detailed comparison between populations. For this abundance analysis, the original sample-level relative abundances were multiplied by 100 and expressed as percentages. At the functional level, 16 KEGG level 3 pathways were selected by combining overall relative abundance with the mean differences between populations, calculating the specific Par–Bis difference. Species and pathway comparisons utilized two-sided Mann–Whitney *U* tests. Benjamini–Hochberg correction was applied separately across the complete sets of tested species and pathways before features were selected for visualization. Statistical significance was determined using adjusted *q* values rather than nominal *p* values, with symbols defined as * *q* < 0.05, ** *q* < 0.01, and *** *q* < 0.001.

### 2.9. Integration of Metagenomic and Metatranscriptomic Profiles and Estimation of Relative Transcriptional Activity

To distinguish the DNA community reservoir from relative transcriptional representation in RNA, we adopted the logic used in paired metagenomic and metatranscriptomic studies, where MT/MG ratios serve as a proxy for relative transcriptional activity [22,23,24]. For a given species or KEGG pathway feature *i*, the relative activity ratio was defined as: log2 activity ratio_i_ = log2 [(RNA relative abundance_i_ + 1 × 10^−6^)/(DNA relative abundance_i_ + 1 × 10^−6^)]. This ratio estimates the extent to which a given taxon or functional pathway is transcribed relative to its own genomic abundance within the same sample. A log_2_ value above zero indicates that RNA representation exceeds the corresponding DNA background (i.e., the taxon or pathway is relatively over-transcribed), whereas a value below zero indicates weaker transcriptional representation than expected from its genomic abundance alone. Importantly, because both MG and MT abundances are compositional relative values, the RNA/DNA ratio functions as a relative enrichment index rather than an absolute measure of transcription per DNA template or per cell. It does not account for taxon-specific differences in genome copy number, ribosomal RNA depletion efficiency, mRNA stability, or the proportion of transcriptionally active cells within a population. These limitations are widely recognized in paired metagenomic–metatranscriptomic investigations [22,24], and the ratio should therefore be interpreted as a comparative indicator of relative transcriptional representation across populations, not as a direct readout of cellular metabolic activity or single-cell gene expression. At the species level, only features with a maximum global relative abundance ≥ 0.001% in MG or MT were retained, yielding 279 shared species. Quadrant analysis used the mean DNA and RNA relative abundances for Bis and Par. Each retained species generated one data point in each population. The 75th percentiles of DNA and RNA relative abundance across all data points were used as common thresholds to classify four states: high DNA/high RNA, high DNA/low RNA, low DNA/high RNA and low DNA/low RNA.

Following the quadrant state classification, RNA/DNA ratios were employed to normalize RNA abundance against its corresponding DNA background, allowing for the comparison of relative transcriptional activity for specific species or KEGG pathways between populations. At the species level, activity ratios were calculated separately for Bis and Par for all retained species. At the functional level, the same procedure was applied to shared KEGG level 3 pathways, with an emphasis on pathways related to cofactor synthesis, adaptation to blood meals, immune interactions, and energy, amino acid, and lipid metabolism. Comparisons between populations were conducted using two-sided Mann–Whitney *U* tests, with Benjamini–Hochberg correction applied separately to *p* values. Features were ranked by FDR significance, nominal significance, and the absolute difference in mean ratios between populations; the five species and KEGG pathways with higher relative activity ratios in each population were subsequently displayed. This ratio is a compositional relative activity index and does not represent absolute microbial load, expression in individual cells or actual metabolic activity [41].

### 2.10. Statistical Analysis

Continuous variables were compared between Bis and Par using two-sided Mann–Whitney U tests. The Wilcoxon rank-sum test reported by the supplier was treated as synonymous with the rank sum test for independent samples. Multiple testing was controlled using the Benjamini–Hochberg false discovery rate procedure [42]. The main text reports raw *p* values, adjusted *q* values, group means and effect directions. A *q* value < 0.05 was established as the significance threshold after adjustment for multiple testing. With only five replicates per population, complete rank separation can produce the same discrete minimum *p* value and the same *q* value; therefore, statistical significance was interpreted together with effect size, sample distributions and analysis level. Except for LEfSe v1.1.0, which used nominal *p* < 0.05 and LDA score > 4.0, unadjusted *p* values were not used as substitutes for conclusions supported after FDR correction.

## 3. Results

### 3.1. Sequencing Quality Control and Microbial Community Diversity

We obtained five MG and five MT datasets for each of the Bis and Par populations. MG clean data ranged from 20.42 to 25.23 Gb per sample, with Q30 values of 91.62–93.99%. The group mean MG clean data were 23.04 Gb for Bis and 22.71 Gb for Par; after host removal, 4.58 Gb and 1.77 Gb remained, corresponding to retained non-host fractions of 19.89% and 7.79%, respectively. MT clean data ranged from 9.65 to 12.20 Gb per sample, with Q30 values of 93.13–94.91%. Group mean MT clean data were 10.22 Gb for Bis and 11.29 Gb for Par, with retained non-host data of 3.30 Gb and 5.16 Gb and retained fractions of 32.31% and 45.70%, respectively. The mapped rates of the MT non-redundant gene set were 97.63% in Bis and 98.93% in Par. Group mean quality control metrics are shown in Table 1, and data for individual samples are shown in Table S1. Although the retained MG non-host fraction was lower in Par than Bis, every MG sample yielded at least 1.65 Gb of non-host data. Because different host reference genomes were used for the two populations, this retention difference is reported as a data characteristic and was not used for inference about population differences.

**Figure 1:**
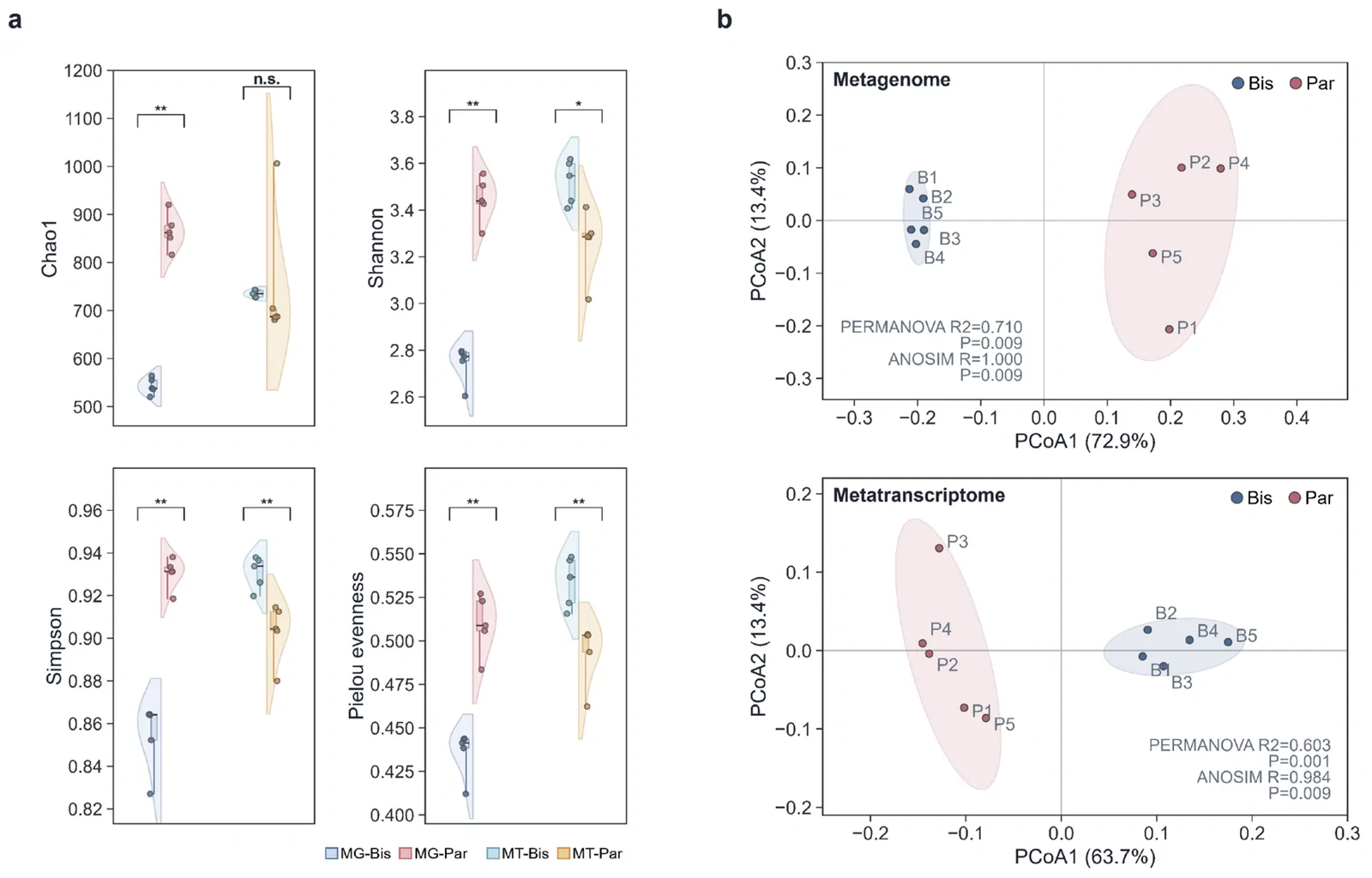
Metagenomic and metatranscriptomic community diversity in bisexual and parthenogenetic *Haemaphysalis longicornis* populations. (**a**) Alpha diversity. Chao1, Shannon, Simpson and Pielou indices for metagenomic and metatranscriptomic communities. Points represent independent biological replicates (*n* = 5 per population); horizontal lines and error bars show the central tendency and dispersion specified in the figure. Comparisons between populations were conducted using two-sided Mann–Whitney *U* tests. (**b**) Beta diversity and community separation. Principal coordinate analysis (PCoA) of metagenomic and metatranscriptomic communities was performed based on Bray–Curtis distances. Shaded ellipses indicate the distribution range for each population; ANOSIM and PERMANOVA statistics are reported within the panels. Bis, bisexual population; Par, parthenogenetic population. All *p* values are from two-sided tests. * *p *< 0.05, ** *p *< 0.01, n.s., not significant.

MG assembly yielded 833,638 non-redundant genes and MT assembly yielded 69,985 non-redundant genes. Alpha diversity showed opposite patterns between the microbial DNA reservoir and the RNA transcript community. In MG profiles, Par exhibited a higher Chao1 index than Bis (865.49 ± 38.00 versus 542.77 ± 17.22), as well as higher Shannon (3.445 ± 0.097 versus 2.744 ± 0.080), Simpson (0.930 ± 0.007 versus 0.854 ± 0.016) and Pielou indices (0.510 ± 0.017 versus 0.436 ± 0.013; all *p* = 0.0079, representing the minimum exact *p*-value for complete rank separation with *n* = 5 per population; Figure 1a). In MT profiles, the Chao1 index did not differ significantly between groups (Bis, 735.14 ± 7.34; Par, 752.97 ± 141.87; *p* = 0.1508), whereas Shannon, Simpson and Pielou indices were higher in Bis than in Par (*p* = 0.0159, 0.0079 and 0.0079, respectively; Figure 1a). Thus, Par harbored a richer microbial DNA reservoir, whereas Bis maintained a more even transcript community at the time of sampling. Given the limited number of biological replicates (*n* = 5 per population), the statistical power to detect small between-group differences may be restricted, and all results should be interpreted accordingly.

Bray–Curtis PCoA showed clustering within each population and separation between populations in both MG and MT profiles (Figure 1b). In MG, ANOSIM *R* = 1.000 (*p* = 0.009) and PERMANOVA *R*^2^ = 0.710 (*F* = 19.59, *p* = 0.009). In MT, ANOSIM *R* = 0.984 (*p* = 0.009) and PERMANOVA *R*^2^ = 0.603 (*F* = 12.18, *p* = 0.001). Thus, differences associated with reproductive form were present not only in the DNA detection profile but also in the RNA transcript community.

### 3.2. Microbial Community Composition Across Reproductive Populations

**Figure 2:**
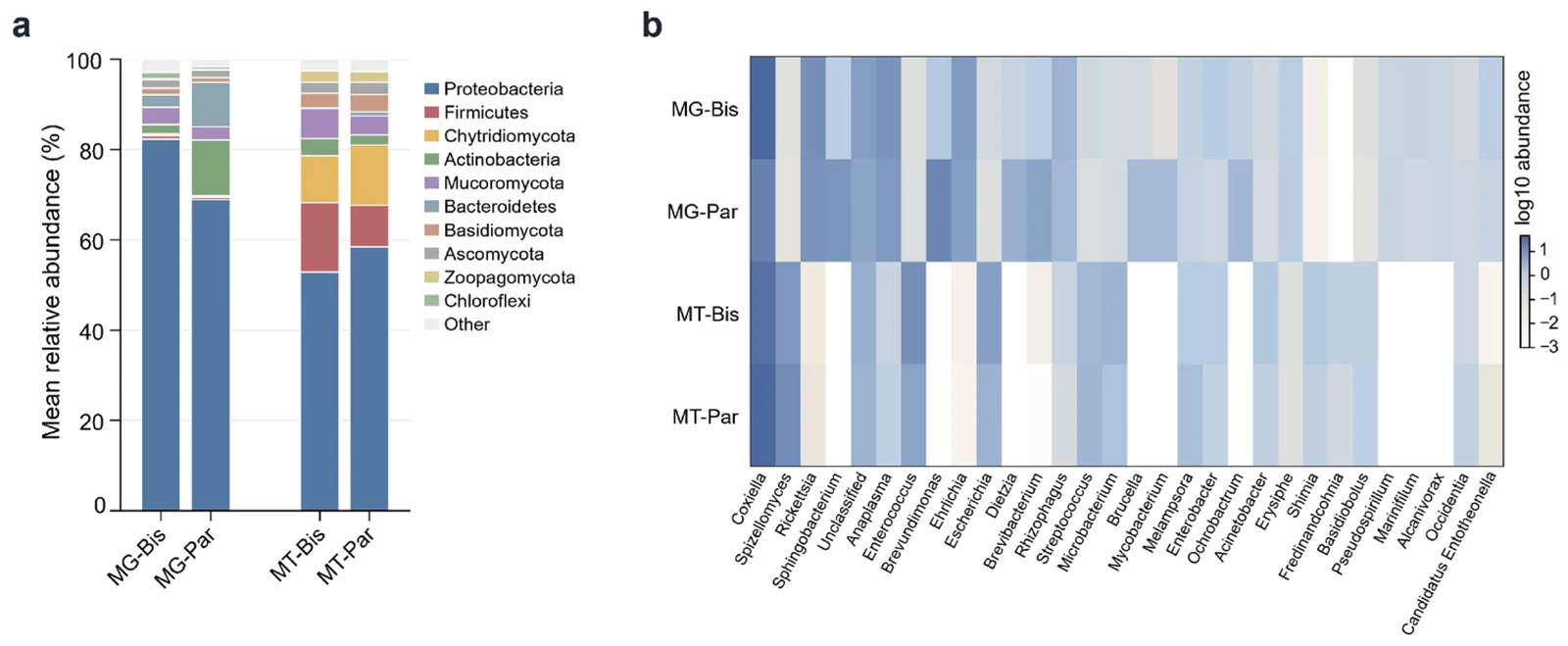
Microbial taxonomic composition of *Haemaphysalis longicornis* bisexual and parthenogenetic populations at the metagenomic and metatranscriptomic levels. (**a**) Phylum composition. Mean relative abundance at the phylum level are presented as stacked bars; taxa with low abundance or taxa not listed separately were combined into the ‘Other’ category. (**b**) Genus composition. The heatmap shows the relative abundance of major genera across both metagenomic and metatranscriptomic samples. Colors indicate relative abundance within each sample, while columns represent independent biological replicates and rows represent taxonomic units. Note that this figure compares relative community composition and does not represent absolute microbial load.

At the phylum level, Proteobacteria dominated both MG and MT profiles, although its relative abundance varied across populations and omics layers (Figure 2a). In MG, Proteobacteria accounted for 82.32% and 68.86% of the group mean relative abundance in Bis and Par, respectively, whereas Actinobacteria (12.41% versus 2.04%) and Bacteroidetes (9.87% versus 2.81%) exhibited higher mean relative abundances in Par. In MT, Proteobacteria remained dominant (Bis, 52.86%; Par, 58.43%), but its relative dominance was weaker than in MG. Firmicutes tended to be more abundant in Bis (15.40% versus 9.19%), whereas Chytridiomycota exhibited a higher mean in Par (13.37% versus 10.35%). These changes indicate that the DNA composition at phylum level of dominant taxa cannot be directly interpreted as their RNA transcript contribution.

At the genus level, *Coxiella* emerged as a shared dominant member, yet it showed opposite population biases in MG and MT (Figure 2b). In MG, *Coxiella* accounted for 43.78% in Bis and 18.02% in Par, whereas in MT it accounted for 33.66% and 45.55%, respectively. Par MG also showed higher mean abundances for *Brevundimonas* (14.87% versus 1.42%), *Sphingobacterium* (8.77% versus 0.98%), *Brevibacterium* (5.57% versus 0.88%), *Dietzia* (3.53% versus 0.52%), *Brucella* annotation signals (2.29% versus 0.19%) and *Mycobacterium* annotation signals (2.30% versus 0.08%). In Bis MT, *Enterococcus* (11.12% versus 5.41%), *Escherichia* (6.17% versus 3.30%) and *Microbacterium* (3.09% versus 1.67%) contributed higher transcript proportions. Thus, the two reproductive populations exhibited distinct genus-level profiles despite a shared symbiotic background dominated by *Coxiella*.

### 3.3. Core Microbial Communities and Signals Related to Viruses, Pathogens and Symbionts

**Figure 3:**
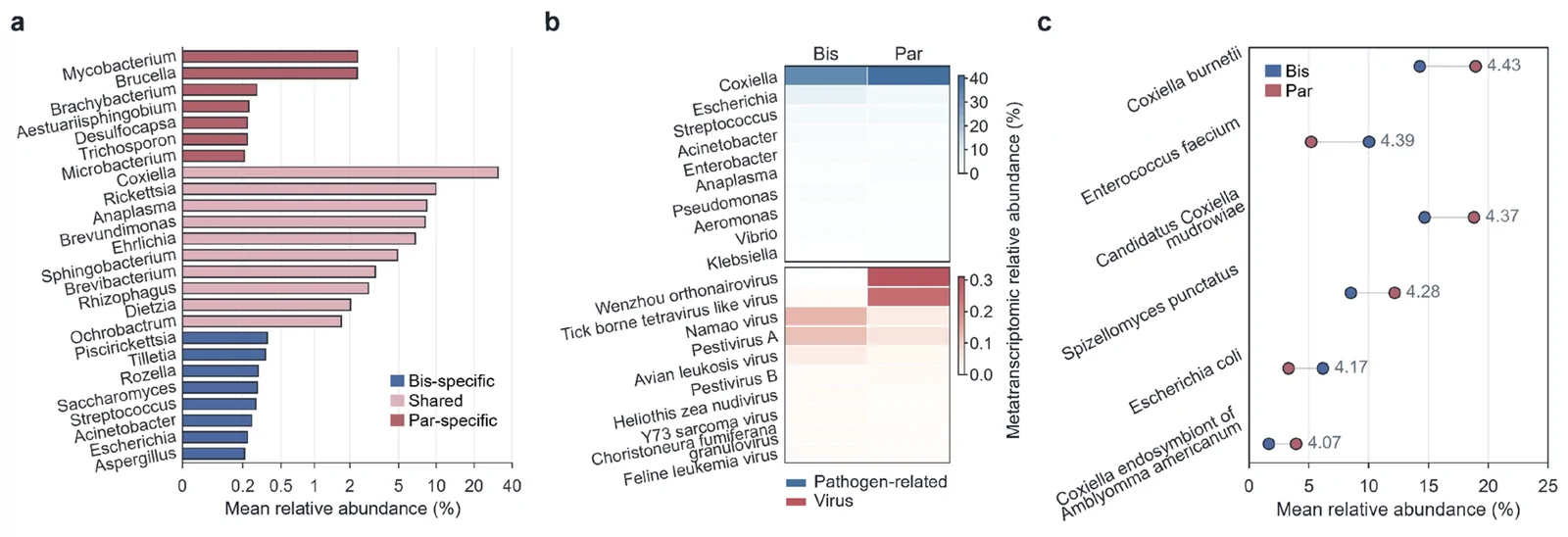
Core metagenomic genera, metatranscriptomic viral signals and candidate markers related to pathogens and symbionts. (**a**) Core genera. Shared genera and population-specific genera were defined from metagenomic data. A core genus was defined as having a relative abundance > 0.001% in at least four of five samples and a group mean relative abundance ≥ 0.2%. (**b**) Viral transcript signals. Mean relative abundance of major annotation entries related to viruses in the metatranscriptomic data. (**c**) Candidate pathogen and symbiont markers. Linear discriminant analysis effect size (LEfSe) markers were identified from the metatranscriptomic abundance matrix at the species level, selected using an LDA score > 4.0 and nominal *p* < 0.05; points represent group mean relative abundance. Species and virus names represent database annotation signals and do not confirm the presence of viable pathogens or transmission capacity.

Using the prespecified prevalence and mean abundance thresholds, Bis and Par shared the core genera *Coxiella*, *Rickettsia*, *Anaplasma*, *Ehrlichia*, *Brevundimonas*, *Sphingobacterium*, *Brevibacterium*, *Rhizophagus*, *Dietzia* and *Ochrobactrum* (Figure 3a). Bis-specific core genera included *Aspergillus*, *Escherichia*, *Acinetobacter*, *Streptococcus*, *Saccharomyces*, *Rozella*, *Tilletia* and *Piscirickettsia*. Par-specific core genera included *Microbacterium*, *Trichosporon*, *Desulfocapsa*, *Aestuariisphingobium*, *Brachybacterium*, *Brucella* and *Mycobacterium*. The overall relative abundance of MT entries related to viruses was low, but several signals showed different patterns between populations (Figure 3b). Wenzhou orthonairovirus and tick-borne tetravirus-like virus had group mean relative abundances of 0.341% and 0.259%, respectively, in Par, whereas the corresponding Bis signals were near zero or lower. Namao virus and Pestivirus A were higher in Bis (0.133% and 0.119%, respectively) than in Par (0.047% and 0.057%). These viral signals were generally low in abundance and should be treated as annotation-level observations, as the present study was not designed to validate active viral infection.

LEfSe analysis further identified directional MT markers (Figure 3c). Par showed higher annotation signals for *Coxiella burnetii* (LDA = 4.432, nominal *p* = 0.0283; Bis 14.28%, Par 18.94%), *Candidatus Coxiella mudrowiae* (LDA = 4.370, *p* = 0.0090; 14.68% versus 18.81%), *Spizellomyces punctatus* (LDA = 4.277, *p* = 0.0090; 8.51% versus 12.18%) and *Coxiella* endosymbiont of *Amblyomma americanum* (LDA = 4.074, *p* = 0.0090; 1.66% versus 3.94%). Bis showed higher *Enterococcus faecium* (LDA = 4.392, *p* = 0.0090; 10.04% versus 5.19%) and *Escherichia coli* (LDA = 4.168, *p* = 0.0090; 6.17% versus 3.30%). Although based on nominal *p*-values, the stringent LDA threshold (>4.0) ensures the robust identification of these discriminative markers. Given that short-read classification of closely related *Coxiella* species and endosymbiotic lineages can be affected by conserved sequences, these names were interpreted as database annotation markers rather than definitive pathogen confirmations. Together, the core genera, viral signals and differential markers indicate consistent differences between the reproductive populations in their symbiotic backgrounds and in potential transcript signatures related to pathogens.

### 3.4. Metagenomic Functional Potential and Differential Annotations

**Figure 4:**
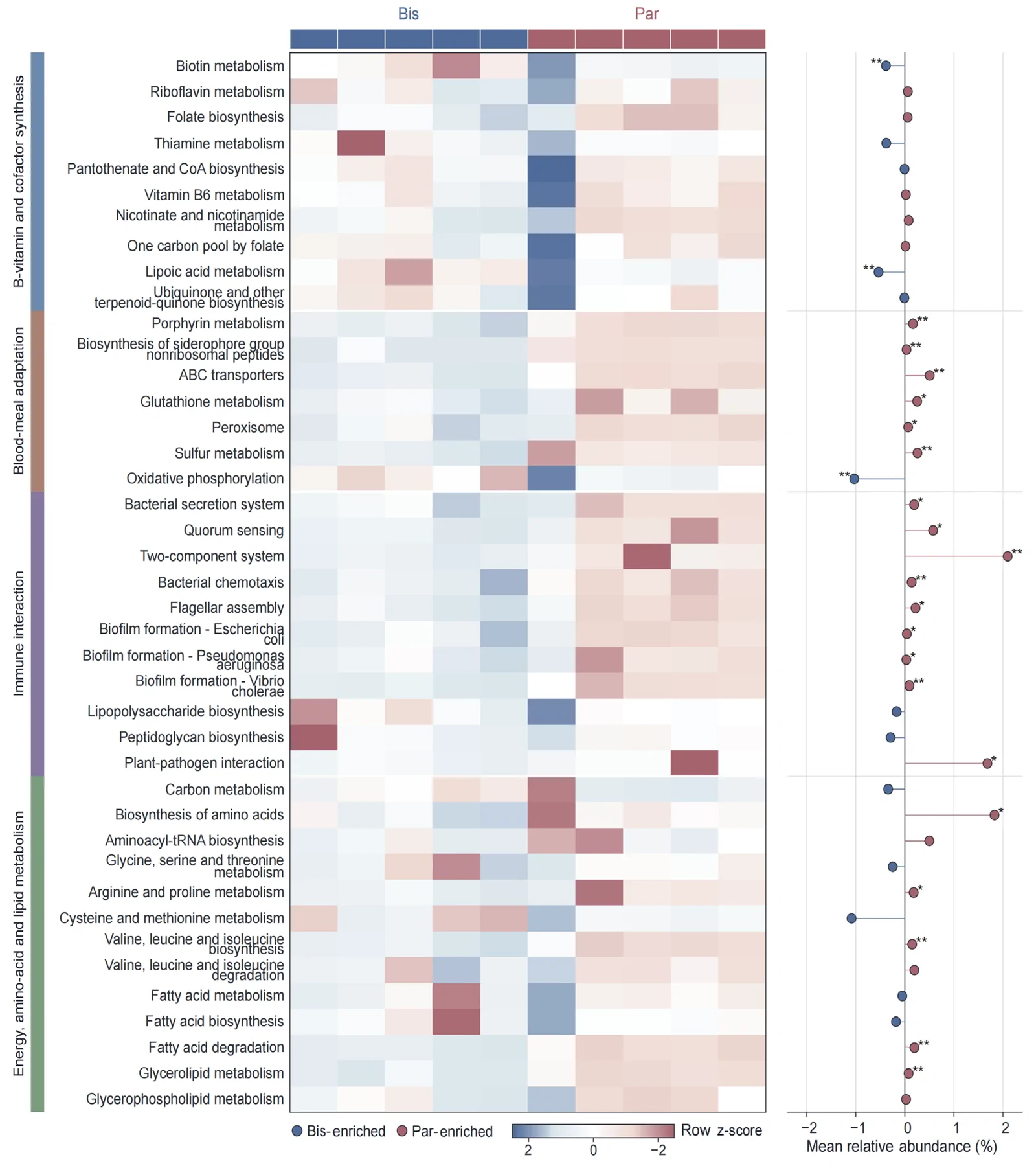
Metagenomic functional potential of KEGG level 3 pathways associated with cofactors, environmental responses and host interactions. The heatmap shows the relative abundance of 41 prespecified KEGG level 3 pathways. Values were transformed as log10 (relative abundance (%) + 1 × 10^−4^) and standardized by pathway using row-wise z-scores calculated by row. Color indicates the relative level of each sample within a given pathway compared to the row (pathway) mean: blue represents a negative z-score (below the mean), red represents a positive z-score (above the mean), and white represents a z-score of zero (equal to the mean); the top annotation indicates Bis and Par. Differences in pathway abundance between populations: The plot shows the difference between Par and Bis group means in percentage points and the direction of change. Comparisons between populations were conducted using two-sided Mann–Whitney *U* tests with Benjamini–Hochberg FDR correction. * *p *< 0.05, ** *p *< 0.01.

The 41 prespecified KEGG level 3 pathways in the metagenomic data showed complementary coding potential between the two populations (Figure 4). Bis had higher biotin metabolism (0.707% versus 0.321%), lipoic acid metabolism (1.286% versus 0.749%) and oxidative phosphorylation (2.144% versus 1.112%; all *p* = 0.0090, *q* = 0.0349). Par had higher porphyrin metabolism (0.212% versus 0.047%), siderophore group nonribosomal peptide biosynthesis (0.0347% versus 0.0002%), ABC transporters (0.755% versus 0.249%), sulfur metabolism (0.293% versus 0.036%), fatty acid degradation (0.262% versus 0.068%), two-component systems (2.448% versus 0.352%), bacterial chemotaxis (0.178% versus 0.039%) and biofilm formation (*Vibrio cholerae*) (0.127% versus 0.035%; all *p* = 0.0090, *q* = 0.0349). Heatmap colours represent pathway-wise row z-scores derived from log10-transformed relative abundances: red and blue indicate values above and below the pathway-specific mean across samples, respectively, whereas white indicates values close to that mean. Colour intensity therefore reflects within-pathway variation rather than absolute abundance differences among pathways. These identical *p* and *q* values are characteristic of discrete non-parametric statistics with small sample sizes and do not imply identical effect sizes across pathways. These identical *p* and *q* values arise because, with only five replicates per group, any pathway showing complete rank separation between Bis and Par reaches the same minimum exact *p* value. They indicate that all listed pathways show the same categorical pattern of group separation, but they do not imply that the magnitude of difference is equivalent across pathways.

Analyses using specialized functional databases identified additional differences in metagenomic annotations (Figure S1). Par was enriched in CAZy families GT2, GT3, CBM21, GH15, GH0, GH13_31, GH78, GT20 and GH23, whereas Bis was enriched in GT51 (all *p* = 0.0090, *q* = 0.0315; GT51: Bis 29.99%, Par 1.94%). In PHI-base, the increased virulence (hypervirulence) annotation category was higher in Par (15.63% versus 2.91%), whereas loss of pathogenicity was higher in Bis (17.39% versus 2.25%; both *p* = 0.0079, *q* = 0.0317). In CARD, Par showed higher annotation categories for peptide, cephalosporin, carbapenem, monobactam and mupirocin-like classes, whereas Bis showed higher phenicol, diaminopyrimidine and fluoroquinolone classes (all *p* = 0.0090, *q* = 0.0150).

### 3.5. Metatranscriptomic Species Composition and Functional Expression

**Figure 5:**
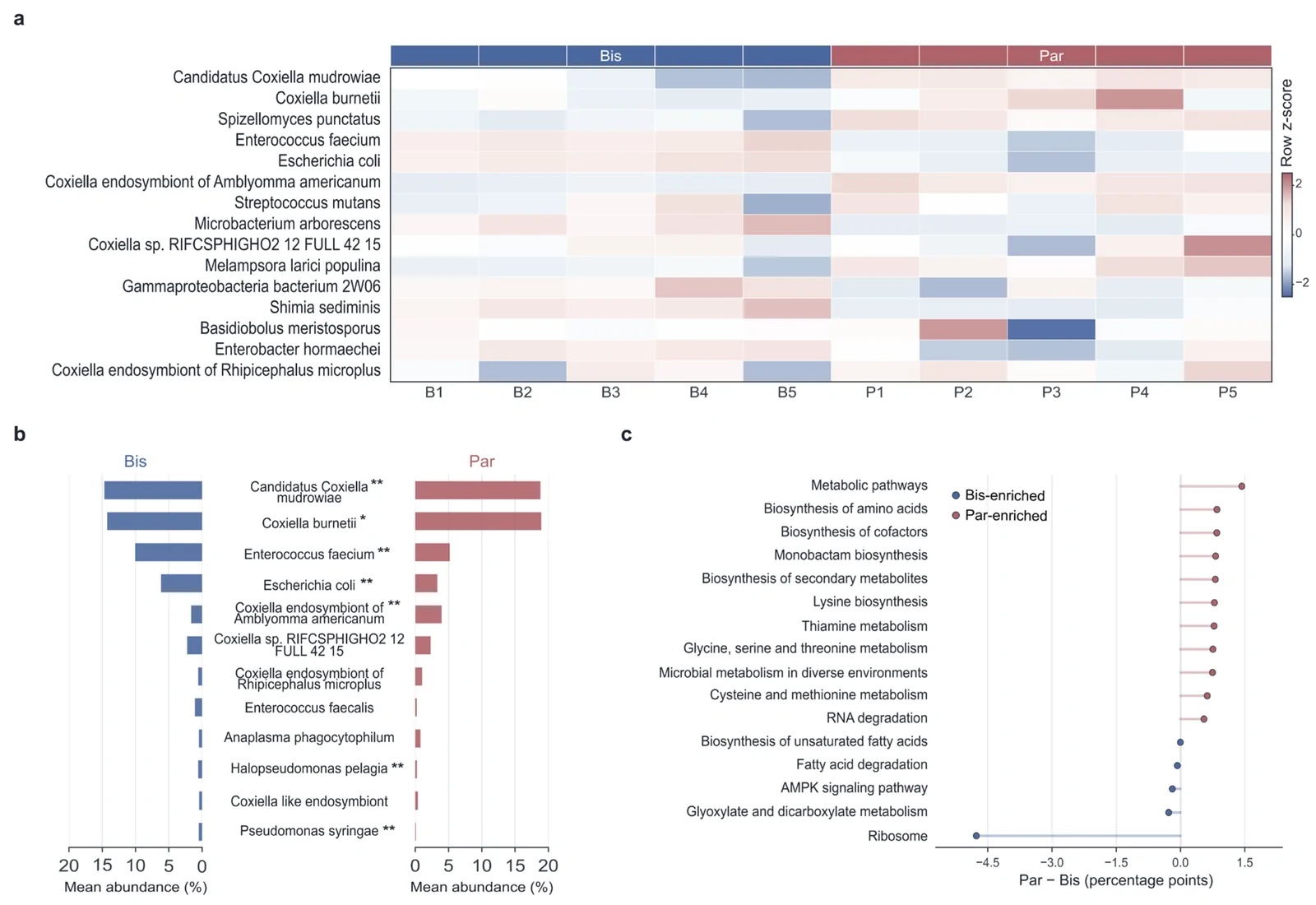
Major species and functional transcript profiles in the metatranscriptome. (**a**) Dominant species. The 15 species are ranked by overall mean relative abundance in the metatranscriptomic data. Values were transformed as log10 (relative abundance (%) + 1 × 10^−4^) and standardized by species using row-wise z-scores; columns represent independent samples. (**b**) Candidate pathogen and symbiont species. Group mean metatranscriptomic relative abundance of the 12 most abundant species in the prespecified candidate set related to pathogens and symbionts. Symbols indicate significant differences after Benjamini–Hochberg correction; taxonomic names represent reference database annotations. (**c**) Metatranscriptomic KEGG pathway differences. Differences between Par and Bis group means in metatranscriptomic relative abundance are shown for 16 prespecified KEGG level 3 pathways. Points represent mean differences and lines indicate the direction of change. Since no pathway reached *q* < 0.05 in this panel, all differences are interpreted as exploratory transcript patterns. Metatranscriptomic relative abundance represents transcript composition and is not equivalent to absolute activity or activity in individual cells. * *p *< 0.05, ** *p *< 0.01.

The 15 MT species with the highest overall mean abundance represented the major transcript sources in the community at the time of sampling (Figure 5a). *Candidatus Coxiella mudrowiae* (Bis 14.68%, Par 18.81%), *Coxiella burnetii* annotation signals (14.28% versus 18.94%) and *Spizellomyces punctatus* (8.51% versus 12.18%) had higher transcript relative abundances in Par, whereas *Enterococcus faecium* (10.04% versus 5.19%), *Escherichia coli* (6.17% versus 3.30%) and *Microbacterium arborescens* (3.09% versus 1.67%) were higher in Bis. The transcript relative abundance of *Coxiella* endosymbiont of *Amblyomma americanum* was approximately 2.4-fold higher in Par than Bis (3.94% versus 1.66%). The Figure 5a heatmap colours have the same meaning as those in Figure 4. Among candidate species related to pathogens and symbionts, *Candidatus Coxiella mudrowiae* and *Coxiella* endosymbiont of *Amblyomma americanum* were higher in Par, whereas *Enterococcus faecium*, *Escherichia coli*, *Halopseudomonas pelagia* and *Pseudomonas syringae* were higher in Bis (all *q* = 0.0431; Figure 5b). Although the *Coxiella burnetii* annotation signal had a higher group mean in Par, the difference reached only nominal significance (*p* = 0.0283, *q* = 0.1051) and was not supported after FDR correction.

Functional transcript analysis revealed that none of the 16 prespecified KEGG level 3 pathways reached statistical significance *q* < 0.05 (Figure 5c). Metabolic pathways, biosynthesis of cofactors, monobactam biosynthesis, biosynthesis of secondary metabolites, lysine biosynthesis and thiamine metabolism showed trends toward higher values in Par. Ribosome, fatty acid degradation, AMPK signaling and glyoxylate and dicarboxylate metabolism showed trends toward higher values in Bis, but none had support after correction for multiple testing.

Differences in specialized MT functional annotations were concentrated within selected CAZy families (Figure S2). GH23 and GT24 transcript relative abundances were higher in Par, whereas GH38, AA1, GT57, GT58, GH72, GT8, PL1 and GT95 were higher in Bis (all *p* = 0.0079, *q* = 0.0388). PHI-base and CARD annotation classes showed directional differences, but most did not reach *q* < 0.05. Overall, metatranscriptomic differences supported after FDR correction were limited mainly to a small number of CAZy families, whereas the prespecified KEGG pathways and PHI-base and CARD classes did not show comparably consistent evidence for differences between populations.

### 3.6. Integrated Metagenomic and Metatranscriptomic Profiles and Relative Transcriptional Activity

**Figure 6:**
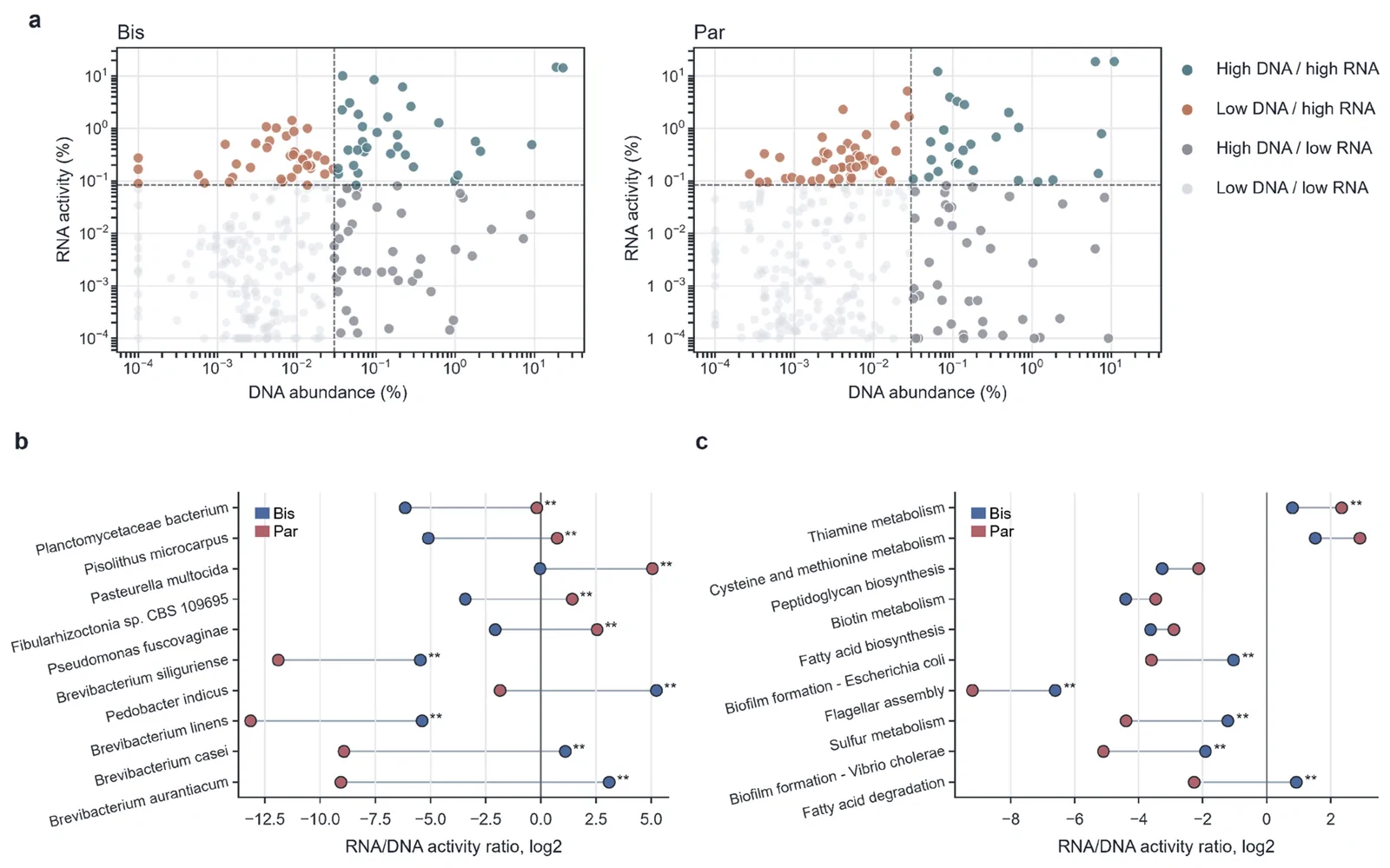
Integrated metagenomic and metatranscriptomic profiles of relative RNA-to-DNA activity at the species and KEGG pathway levels. (**a**) DNA and RNA abundance states. Group mean DNA and RNA relative abundances of 279 shared species in Bis and Par. Dashed lines represent the 75th percentile thresholds across all 558 points representing each species in each population (DNA = 0.029671%; RNA = 0.083003%), defining high DNA/high RNA, high DNA/low RNA, low DNA/high RNA and low DNA/low RNA states. (**b**) Differences in species activity ratios. Displays the species with the largest differences in RNA/DNA activity ratios between Bis and Par. (**c**) Differences in pathway activity ratios. Highlights representative KEGG level 3 pathways with distinct RNA/DNA activity ratios. The activity ratio was defined as log2 [(RNA relative abundance + 1 × 10^−6^)/(DNA relative abundance + 1 × 10^−6^)]; points represent group means and connecting lines indicate the direction of shift for the same feature. Two-sided Mann–Whitney *U* tests were followed by Benjamini–Hochberg FDR correction. Several top-ranked species (e.g., Planctomycetaceae bacterium, *Pisolithus microcarpus*, *Fibularhizoctonia* sp. CBS 109695) are environmental database assignments with no established biological association with ticks; their inclusion reflects compositional ratio statistics rather than confirmed tick–microbe interactions. ** *p *< 0.01.

A total of 279 shared species features in the metagenomic and metatranscriptomic datasets were matched. Using Bis and Par group means, this yielded 558 data points representing each species within each population (Figure 6a). Under the common 75th percentile thresholds, 59 points were high DNA/high RNA, 81 were high DNA/low RNA, 81 were low DNA/high RNA and 337 were low DNA/low RNA. The corresponding counts were 33, 41, 37 and 168 for Bis and 26, 40, 44 and 169 for Par. The low DNA/high RNA category was slightly more frequent in Par, suggesting that some low-DNA members had stronger relative RNA representation.

At the species level, RNA/DNA activity ratios identified candidate members that differed from those highlighted by MT abundance alone (Figure 6b). Par showed higher ratios for Planctomycetaceae bacterium (ΔPar-Bis = 5.962, *q* = 0.0483), *Pisolithus microcarpus* (Δ = 5.833, *q* = 0.0403), *Pasteurella multocida* (Δ = 5.090, *q* = 0.0403), *Fibularhizoctonia* sp. CBS 109695 (Δ = 4.847, *q* = 0.0403) and *Pseudomonas fuscovaginae* (Δ = 4.612, *q* = 0.0403). Bis showed higher ratios for *Brevibacterium aurantiacum* (Δ = −12.158, *q* = 0.0403), *Brevibacterium casei* (Δ = −10.022, *q* = 0.0403), *Brevibacterium linens* (Δ = −7.767, *q* = 0.0403), *Pedobacter indicus* (Δ = −7.086, *q* = 0.0403) and *Brevibacterium siliguriense* (Δ = −6.440, *q* = 0.0403). Notably, Planctomycetaceae bacterium, *Pisolithus microcarpus*, and *Fibularhizoctonia* sp. CBS 109695 among the top-ranked Par-enriched entries are environmental taxa with no established association with *H. longicornis*; these entries reflect database annotations rather than confirmed tick–microbe interactions. These features did not necessarily have the highest RNA abundance; rather, their RNA relative abundance was higher than expected from their own DNA background, indicating that the two reproductive populations differed not only in the members they carried but also in those exhibiting higher relative transcriptional activity.

At the KEGG pathway level, the RNA/DNA activity ratio for thiamine metabolism was higher in Par than Bis (2.341 versus 0.807, Δ = 1.533, *p* = 0.0079, *q* = 0.0426; Figure 6c). Cysteine and methionine metabolism (Δ = 1.397, *q* = 0.349), peptidoglycan biosynthesis (Δ = 1.140, *q* = 0.540) and biotin metabolism (Δ = 0.935, *q* = 1.000) showed trends toward higher values in Par but did not pass FDR correction. In Bis, biofilm formation (*Escherichia coli*), flagellar assembly, sulfur metabolism, biofilm formation (*Vibrio cholerae*) and fatty acid degradation had higher RNA/DNA activity ratios (all *q* = 0.0426). Overall, the RNA/DNA analysis referenced to DNA identified relative transcriptional activity differences in selected microbial members and functional pathways between the two populations.

## 4. Discussion

Whether reproductive strategy is accompanied by ecological differentiation of microbial communities between populations is important for understanding the biology and potential adaptation of *H. longicornis*. Here, Bis and Par differed in community diversity, taxonomic composition and core members. Par had higher MG richness, diversity and evenness, whereas Bis had higher MT alpha diversity indices, and the two populations separated in both MG and MT community space. This pattern suggests that Par harbored a broader microbial DNA reservoir, whereas the members contributing RNA in Bis were more evenly distributed at the sampling time point; it does not indicate that either population had globally greater microbial activity. The discordance between MG and MT diversity further shows that the DNA community reservoir cannot be used as a direct proxy for the RNA transcript community, consistent with paired MG and MT studies [22,23,24]. Proteobacteria dominated both populations, and *Coxiella* was a shared dominant genus, but its relative abundance was higher in Bis MG and Par MT, indicating that DNA reservoir size and RNA transcript contribution were not directly coupled. The two populations shared core genera including *Coxiella*, *Rickettsia*, *Anaplasma* and *Ehrlichia*, while also retaining reproducibly detected population-specific core members. Previous studies have documented differences between Bis and Par in ploidy, biological traits and vector phenotypes [9,10], while ovarian integrated omics and population genetic analyses provide further evidence of molecular and genetic differentiation associated with reproductive lineage [43,44]. Nevertheless, tick microbiome composition can also vary with tick characteristics, blood meal and geographic background [45,46]; the differences observed here should therefore be interpreted as patterns observed in these populations under the present laboratory conditions rather than universal signatures of the two reproductive forms. Both laboratory populations had been reared on New Zealand White rabbits for over 10 generations prior to this study, which may have selected for or against certain microbial taxa compared with field-collected ticks. Continuous feeding on a single host species could also have simplified the microbiome by filtering out environmental microbes acquired from alternative hosts or habitats. The Bis and Par populations were maintained under identical rearing conditions, which reduces but does not eliminate the possibility that lab adaptation history contributed to the observed differences. Untargeted tick metatranscriptomics can identify diverse bacterial, protozoan and viral candidates [47,48]. However, the low-abundance viral transcripts and annotations related to pathogens or symbionts detected here should remain screening leads because short-read assignments alone cannot establish infection, replication or transmission competence [49,50].

Functional analyses showed that MG coding potential and MT transcript composition differed in scope between the two populations. Par had higher MG potential in environmental sensing, membrane transport, chemotaxis, biofilm formation, and porphyrin metabolism and iron acquisition, whereas Bis had higher potential for biotin metabolism, lipoic acid metabolism and oxidative phosphorylation. These patterns suggest complementary functional reservoirs related to environmental response, resource exchange, cofactor synthesis and energy metabolism. CAZy, PHI-base and CARD annotations added information on carbohydrate-active enzymes, genes involved in host interactions and homologues associated with resistance, but these categories represent relative composition based on annotations and cannot be interpreted directly as substrate utilization, virulence phenotypes or clinical antimicrobial resistance [37,39,40]. At the MT level, differences supported after FDR correction were concentrated in a limited number of species and CAZy families, whereas the prespecified KEGG pathways and most PHI-base and CARD classes showed no stable significant differences. Thus, the RNA signal was selective rather than indicative of broad restructuring of the functional transcriptome, and functional potential encoded in DNA was not necessarily transcribed in the same direction at the sampling time point. *Coxiella*-like endosymbionts and their B vitamin biosynthetic functions have been linked to nutritional compensation and tick reproductive fitness [14,51,52]. Functional studies have further shown that disruption of a *Coxiella* endosymbiont can alter tick physiological programs and impair blood-feeding capacity [53], while transcriptomic analysis of *H. longicornis Coxiella*-like endosymbionts revealed abundant expression of genes involved in B vitamin biosynthesis in the ovaries and Malpighian tubules [54]. These findings strengthen the biological plausibility of the distinct *Coxiella* profiles and the differences in biotin, lipoic acid and thiamine metabolism, but do not establish the responsible symbiont lineage or a causal effect on host reproduction. The inverse distribution of Coxiella across the MG and MT layers further illustrates this decoupling. Coxiella was more abundant in Bis than in Par at the MG level (43.78% versus 18.02%), but showed higher relative abundance in Par at the MT level (45.55% versus 33.66%). This pattern may reflect strain-level differences between the two populations or differences in the proportion of actively transcribing cells. The elevated thiamine metabolism RNA/DNA ratio in Par is consistent with increased Coxiella transcriptional activity relative to its DNA load. Given the known role of Coxiella-like endosymbionts in B vitamin provisioning, this raises the possibility that parthenogenetic ticks maintain a smaller but more transcriptionally active Coxiella population to support reproductive nutrition, though this requires strain-resolved validation. Experimental SFTSV infection has also been shown to alter the gut bacterial composition of *H. longicornis* [55], indicating that future validation should consider possible interactions between viral status, bacterial community structure and functional transcription.

RNA/DNA integration added information that was not available from MG or MT abundance comparisons alone. MG describes the microbial and functional reservoir, MT describes transcript composition at the sampling time point, and the RNA/DNA ratio evaluates RNA representation relative to the corresponding DNA background [22,23,24]. The quadrant analysis first classified DNA and RNA abundance states among 279 shared species, after which RNA/DNA ratios quantified differences between populations in relative activity. Par showed higher relative activity ratios for thiamine metabolism and several species, whereas Bis showed higher ratios for multiple *Brevibacterium* species and for biofilm formation, flagellar assembly, sulfur metabolism and fatty acid degradation. These features did not necessarily have the highest MT relative abundance; rather, their RNA representation was greater relative to their own DNA reservoir. Thiamine metabolism was only near the significance threshold in the analysis of MT alone but reached the FDR threshold after normalization to the DNA background, indicating that population differences involved not only transcript composition but also the extent of RNA representation relative to genomic reserve. Because RNA/DNA ratios are derived from relative abundance data, they remain compositional and should not be interpreted as measures of absolute microbial load [41]. Benchmarking of amplicon sequencing based on RNA further indicates that community profiles derived from RNA should not automatically be interpreted as quantitative measures of metabolic activity in complex communities [56]. With these limitations in mind, RNA/DNA integration complements analysis of individual omics layers and provides a focused integrated omics approach for prioritizing candidate active members and functional pathways in tick microbiome research.

Taken together, Bis and Par *H. longicornis* populations differed across the microbial reservoir, transcript composition, functional coding potential and RNA/DNA relative activity, although the direction and breadth of these differences varied across analytical layers. By integrating MG, MT and RNA/DNA analyses, this study distinguished the microbial and functional reservoir carried by the community from its RNA contribution at the sampling time point and from RNA representation relative to the corresponding DNA background, identifying *Coxiella* lineages and B vitamin metabolism as candidates for further validation. The contribution of this work lies in the paired MG–MT comparative design and the integrated RNA/DNA analytical framework, rather than in the discovery of previously unreported microbial taxa or functional pathways in *H. longicornis*. This study has three main limitations. First, one laboratory population represented each reproductive form, so the observed microbiome differences cannot be attributed solely to reproductive mode and may also reflect population-specific genetic or environmental background. Throughout the text, comparisons between Bis and Par should be interpreted as population-level differences rather than definitive effects of reproductive strategy per se. Second, only five biological replicates were included per population, which may reduce the power to detect weak differences and limit generalization beyond these laboratory populations. Third, short-read sequencing has limited resolution for closely related pathogens, symbiont lineages and strains, and some taxonomic assignments therefore require cautious interpretation. Larger independent cohorts, quantitative assays targeting individual species and long-read sequencing will be required to validate the principal findings and improve the identification of key microbial candidates, particularly through rigorous in vivo functional validations to definitively elucidate the specific roles of *Coxiella* symbionts in host reproductive development. This study therefore provides a combined metagenomic and metatranscriptomic approach for resolving microbiome differences between populations in *H. longicornis* and establishes a basis for testing how microbial symbiosis and nutritional metabolism may contribute to reproductive adaptation and vector phenotypes.

## Figures and Tables

**Table 1:** Summary of metagenomic and metatranscriptomic sequencing quality control data from bisexual and parthenogenetic populations.

Dataset	Group	Clean Data (Mbp)	No-Host Clean (Mbp)	Reads (Million)	Clean Q20 (%)	Clean Q30 (%)	Clean GC (%)	Effective (%)	Mapped (%)
Metagenome (DNA)	Bis	23,043.47	4582.47	151.24	97.18	92.52	46.88	19.886	87.31
	Par	22,714.01	1770.28	148.98	97.74	93.81	47.26	7.794	76.08
Metatranscriptome (RNA)	Bis	10,224.74	3303.59	67.12	98.13	94.77	55.78	32.310	97.63
	Par	11,286.40	5157.96	73.04	97.28	93.36	56.12	45.701	98.93

## Data Availability

All raw metagenomic and metatranscriptomic sequencing data have been deposited in the NCBI Sequence Read Archive under BioProject PRJNA1513779 (SRA accessions: SRR40540931–SRR40540940 and SRR40557592–SRR40557601). Numerical source data and analysis scripts supporting the findings of this study are openly available on Figshare at doi: 10.6084/m9.figshare.33268200, to facilitate result reproducibility.

## Supplementary Materials

The following supporting information can be downloaded at https://www.mdpi.com/article/10.3390/microorganisms14102345/s1. Figure S1: CAZy, PHI-base, and CARD annotation profiles in the metagenomic data. From top to bottom, the panels show the mean relative abundance (%) of CAZy families, PHI-base phenotype categories and CARD/ARO resistance classes in Bis and Par (*n* = 5 per population). Points and connecting lines show group means for the same annotation category. Comparisons between populations were conducted using two-sided Mann–Whitney *U* tests with Benjamini–Hochberg FDR correction. Asterisks indicate FDR-adjusted significance (** *q* < 0.01); Figure S2: CAZy, PHI-base, and CARD annotation profiles in the metatranscriptomic data. From top to bottom, the panels show mean MT relative abundance (%) of CAZy families, PHI-base phenotype categories and CARD/ARO resistance classes in Bis and Par (*n* = 5 per population). Points and connecting lines show group means for the same annotation category. PHI-base entries containing multiple phenotype terms were divided equally among the corresponding categories before aggregation. Comparisons between populations were conducted using two-sided Mann–Whitney *U* tests with Benjamini–Hochberg FDR correction. Asterisks indicate FDR-adjusted significance (* *q* < 0.05 and ** *q* < 0.01 ). Table S1: Sample-level quality control statistics for the metagenomic and metatranscriptomic datasets.
