# Supplementary material for: Integrated Metagenomic and Metatranscriptomic Analysis Reveals Microbiome Differences Between Bisexual and Parthenogenetic Populations of Haemaphysalis longicornis
Source: Microorganisms. 2026 Oct 14;14(10):2345. doi: 10.3390/microorganisms14102345 (PMC13707428; doi:10.3390/microorganisms14102345)
Supplement: Supplementary file 1 [file microorganisms-14-02345-s001.zip › microorganisms-4589918-supplementary.pdf]

Supplementary Figures and Table

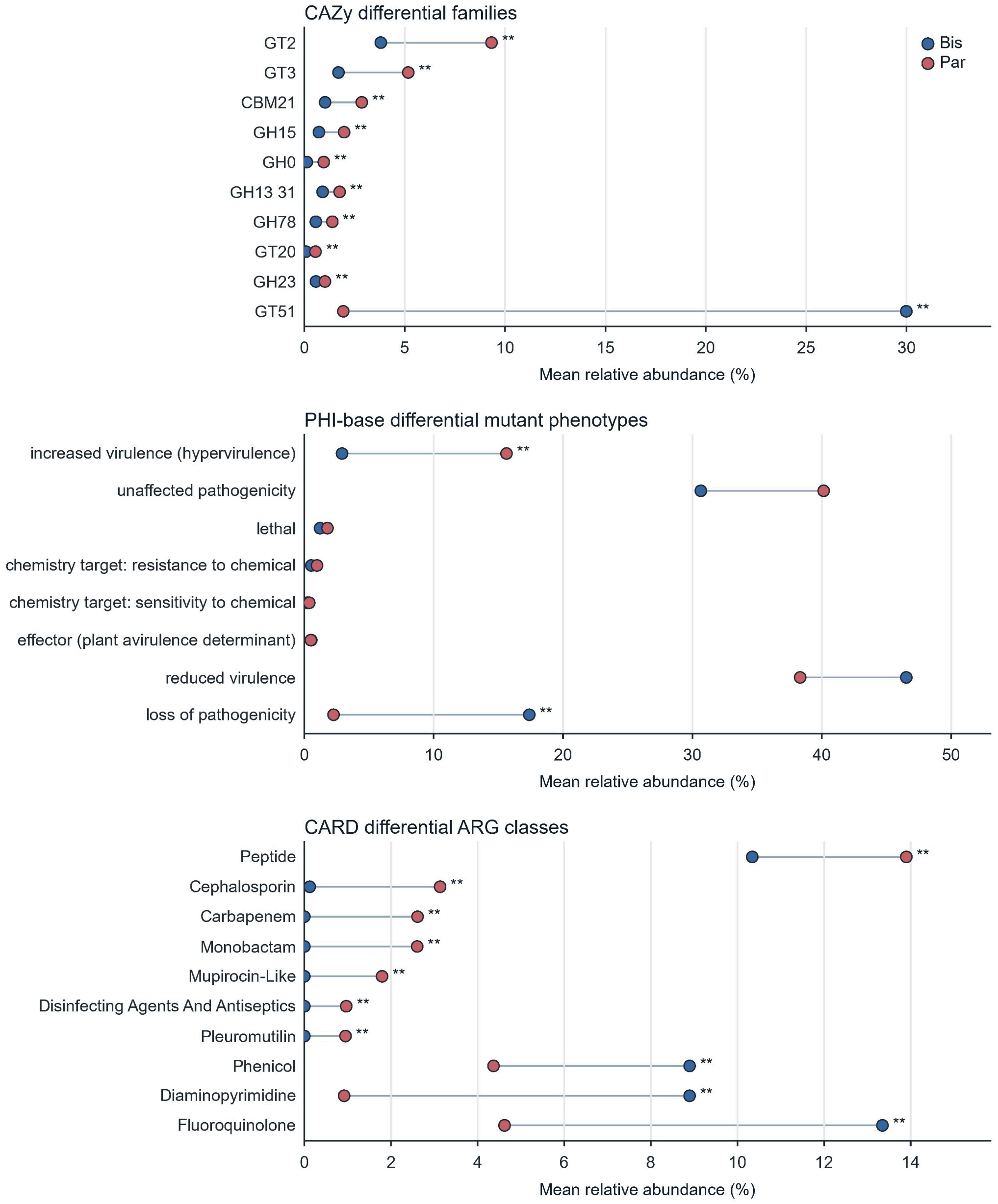

Figure S1. CAZy, PHI-base, and CARD annotation profiles in the metagenomic data. From top to bottom, the panels show the mean relative abundance (%) of CAZy families, PHI-base phenotype categories and CARD/ARO resistance classes in Bis and Par (n = 5 per population). Points and connecting lines show group means for the same annotation category. Comparisons between populations were conducted using two-sided Mann-Whitney U tests with Benjamini-Hochberg FDR correction. Asterisks indicate FDR-adjusted significance (**$q < 0.01$).

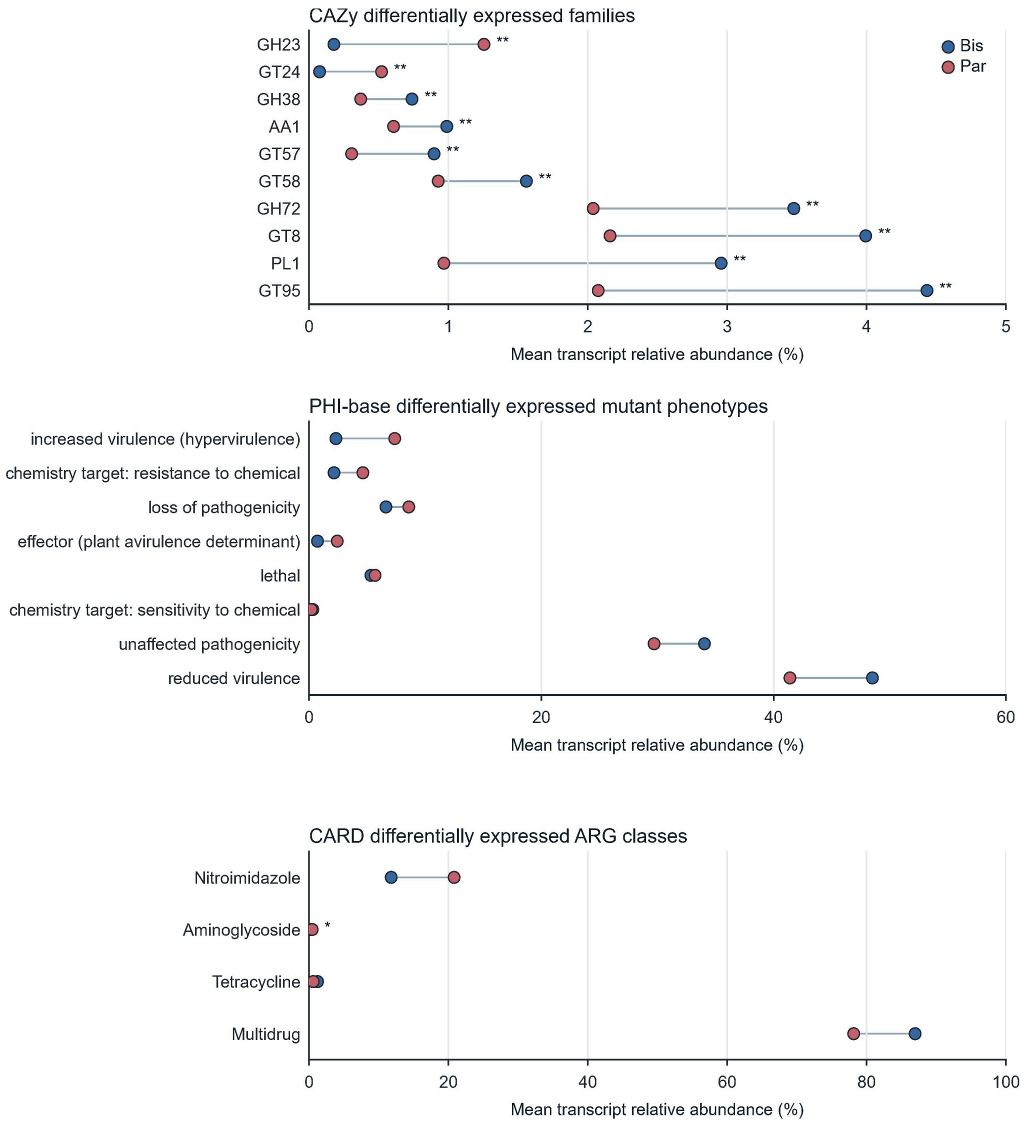

Figure S2. CAZy, PHI-base, and CARD annotation profiles in the metatranscriptomic data. From top to bottom, the panels show mean MT relative abundance (%) of CAZy families, PHI-base phenotype categories and CARD/ARO resistance classes in Bis and Par (*n* = 5 per population). Points and connecting lines show group means for the same annotation category. PHI-base entries containing multiple phenotype terms were divided equally among the corresponding categories before aggregation. Comparisons between populations were conducted using two-sided Mann-Whitney *U* tests with Benjamini-Hochberg FDR correction. Asterisks indicate FDR-adjusted significance (*$q < 0.05$ and **$q < 0.01$).

Table S1. Sample level quality control statistics for the metagenomic and metatranscriptomic datasets.

Omics	Sample	Group	Clean (Gb)	No-host (Gb)	Retained (%)	Reads (M)	GC (%)	Q20 (%)	Q30 (%)	Mapped (%)
Metagenome	B1	Bis	25.23	5.06	20.06	165.49	47.01	97.69	93.71	87.59
Metagenome	B2	Bis	23.27	4.6	19.77	152.64	47.05	97.7	93.73	86.22
Metagenome	B3	Bis	22.93	4.48	19.54	150.55	46.79	96.8	91.62	89.43
Metagenome	B4	Bis	23.37	4.71	20.15	153.36	46.74	96.91	91.86	88.23
Metagenome	B5	Bis	20.42	4.06	19.88	134.14	46.83	96.82	91.67	85.08
Metagenome	P1	Par	22.25	1.86	8.36	145.64	47.48	97.81	93.99	76.45
Metagenome	P2	Par	23.54	1.72	7.31	154.34	47.1	97.68	93.64	75.57
Metagenome	P3	Par	23.19	1.79	7.72	152.24	47.28	97.8	93.94	76.31
Metagenome	P4	Par	22.34	1.65	7.39	146.63	47.22	97.65	93.57	76.04
Metagenome	P5	Par	22.26	1.84	8.27	146.05	47.2	97.78	93.91	76.01
Metatranscriptome	B1	Bis	10.42	3.56	34.17	68.27	55.89	98.03	94.55	97.91
Metatranscriptome	B2	Bis	11.42	3.59	31.44	74.91	55.82	98.2	94.9	97.53
Metatranscriptome	B3	Bis	9.65	3.28	33.99	63.36	55.89	98.2	94.91	97.85
Metatranscriptome	B4	Bis	9.73	2.92	30.01	63.9	55.54	98.09	94.62	97.4
Metatranscriptome	B5	Bis	9.91	3.18	32.09	65.17	55.78	98.15	94.86	97.47
Metatranscriptome	P1	Par	11.88	5.77	48.57	76.44	56.03	97.53	93.73	98.74
Metatranscriptome	P2	Par	11.09	5.04	45.45	71.66	56.07	97.24	93.13	98.95
Metatranscriptome	P3	Par	12.2	5.55	45.49	79.02	56.03	97.14	93.16	98.98
Metatranscriptome	P4	Par	11.16	4.97	44.53	72.72	56.22	97.24	93.56	99.02
Metatranscriptome	P5	Par	10.1	4.46	44.16	65.39	56.26	97.26	93.24	98.95
